# A case report on the physiological responses to extreme heat during Sicily's July 2023 heatwave

**DOI:** 10.14814/phy2.16107

**Published:** 2024-06-07

**Authors:** Davide Filingeri, Alessandro Valenza, Salvatore Ficarra, Victoria Filingeri, Peter R. Worsley, Antonino Bianco

**Affiliations:** ^1^ ThermosenseLab, Skin Sensing Research Group, School of Health Sciences The University of Southampton Southampton UK; ^2^ Sport and Exercise Sciences Research Unit, Department of Psychology, Educational Science and Human Movement University of Palermo Palermo Italy; ^3^ Psychological and Behavioural Sciences, School of Psychology, College of Health, Psychology and Social Care University of Derby Derby UK; ^4^ PRESSURELAB, Skin Sensing Research Group, School of Health Sciences The University of Southampton Southampton UK

**Keywords:** heart rate, heatwave, hot temperature, hyperthermia, skin

## Abstract

July 2023 has been confirmed as Earth's hottest month on record, and it was characterized by extraordinary heatwaves across southern Europe. Field data collected under real heatwave periods could add important evidence to understand human adaptability to extreme heat. However, field studies on human physiological responses to heatwave periods remain limited. We performed field thermo‐physiological measurements in a healthy 37‐years male undergoing resting and physical activity in an outdoor environment in the capital of Sicily, Palermo, during (July 21; highest level of local heat‐health alert) and following (August 10; lowest level of local heat‐health alert) the peak of Sicily's July 2023 heatwave. Results indicated that ~40 min of outdoor walking and light running in 33.8°C Wet Bulb Globe Temperature (WBGT) conditions (July 21) resulted in significant physiological stress (i.e., peak heart rate: 209 bpm; core temperature: 39.13°C; mean skin temperature: 37.2°C; whole‐body sweat losses: 1.7 kg). Importantly, significant physiological stress was also observed during less severe heat conditions (August 10; WBGT: 29.1°C; peak heart rate: 190 bpm; core temperature: 38.48°C; whole‐body sweat losses: 2 kg). These observations highlight the physiological strain that current heatwave conditions pose on healthy young individuals. This ecologically‐valid empirical evidence could inform more accurate heat‐health planning.

## 1 INTRODUCTION

July 2023 has been recently confirmed as Earth's hottest month on record (World Meteorological Organization, 2023a). During this month, extraordinary heatwaves hit parts of southern Europe, including the island of Sicily (Italy), which experienced a significant heat dome during the last 10 days of July (Climate Copernicus, 2023). Sicily's July heatwave was accompanied by record‐breaking temperatures (e.g., the capital Palermo recorded its highest temperature of 47°C [INAF, 2023] and severe wildfires [Reuters, 2023]). Unfortunately, this event aligns with Sicily’ s reputation as the “furnace of Europe” (e.g., the island holds the temperature record for Europe, i.e., 48.8°C) (World Meteorological Organization, 2023b).

Heat waves are the extreme weather events with the highest impact in terms of attributable counts of death (Perkins‐Kirkpatrick & Lewis, 2020). A recent analysis of the heat‐related mortality during Europe's 2022 summer heatwave estimated 61,672 heat‐related deaths, with Italy sharing the highest proportion of mortality numbers and rates (Ballester et al., 2023). Given the extraordinary heat of Europe's 2023 summer heatwave, it is reasonable to expect that such unacceptable levels of heat‐related mortality may become a summer trend for Europe; unless urgent strengthening of existing heat surveillance platforms, prevention plans, and long‐term adaptation strategies occur (Ballester et al., 2023).

The success of heat‐protection planning is strongly dependent on the availability of robust and realistic projections of future heat‐related health risks rooted in sound physiological evidence on human responses to extreme heat (Ebi et al., 2021; Vanos et al., 2020) and humidity (Baldwin et al., 2023). However, such projections remain limited due to the complexity of the physiological and behavioral factors that ultimately determine human health under heat stress (Vanos et al., 2020).

Recently, climate chamber studies performed in young, healthy adults indicated that the predicted limits to human adaptability to extreme heat (i.e., a wet bulb temperature of 35°C) may be significantly lower (i.e., ~30–31°C) and more region‐specific than previously theorized, an observation that questions the accuracy of current heat‐protection planning (Vecellio et al., 2022). Yet, and while important, climate chamber studies with human participants have their limitations. For example, limited solar radiation and airflow in climate chambers result in testing conditions that deviate substantially from ecologically valid, outdoor heat‐stress scenarios experienced by individuals exercising and working during heatwaves (Vecellio et al., 2022). In this respect, field data collected during heatwave periods could add important evidence to further improve our predictions of human adaptability to extreme heat and inform public health policy. However, such field studies remain limited due to their intrinsic challenges (Katavoutas et al., 2009).

To fill this knowledge gap, we took advantage of the extraordinary July 2023 heatwave in southern Europe and performed a series of thermo‐physiological measurements in a healthy young male undergoing resting and physical activity cycles in an outdoor environment in the capital of Sicily, Palermo, during and following the peak of the heatwave.

## 2 MATERIALS AND METHODS

A healthy, white European, 37‐years male (height: 1.75 m; body mass: 82.8 kg; *V*O_2max_: 45 mL/min/kg) performed rest and physical activity cycles on two separate testing days, that is, July 21 and August 10, 2023, while wearing running shorts and shoes only. July 21 was associated with the highest level of heat‐health alert issued by Sicily's department for civil protection (level 3, conditions requiring preventive measures for vulnerable groups; Regione Siciliana, 2023a), whereas August 10 was associated with the lowest alert (level 0, meteorological conditions that do not pose risks to the health of the population; Regione Siciliana, 2023b). The participant is originally from Sicily, although he has been a long‐term resident of the UK (>10 years). He had arrived in Sicily on July 19, that is, 2 days prior to the first testing day, and he therefore presented minimal heat acclimation. Following on the first test, the participant continued to sojourn in Sicily until August 29, during which time he had relatively minor exposure to air‐conditioned environments (i.e., during car journeys only).

**FIGURE 1:**
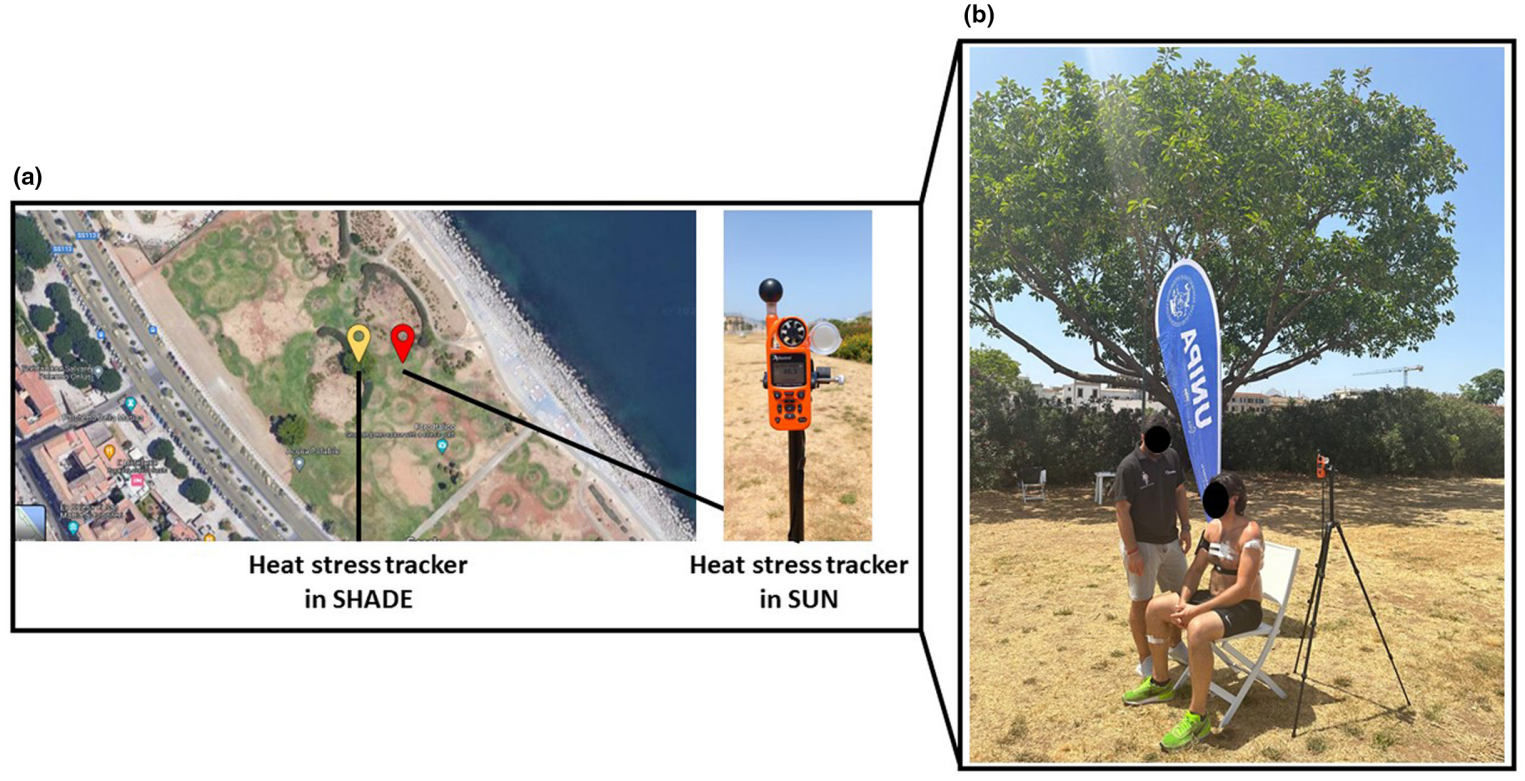
An overview of the study location and setup. The field study location (a) Foro Italico Umberto I, (90133 Palermo, Italy), where the heat stress trackers were placed to monitor shaded and sunlight conditions during resting (b) and light exercise.

The protocol consisted of: (a) 15‐min sitting in a tree‐shaded area; (b) 15‐min of sitting under direct sunlight; (c) 15‐min of walking under direct sunlight; (d) 26‐min of light‐running under direct sunlight; (e) 15‐min of sitting under direct sunlight. All testing occurred in Palermo, in a popular location for recreational exercise (Foro Italico Umberto I, 90133 Palermo, Italy, Figure 1a) between the peak hours of heat (i.e., 12:00–15:00). Our protocol was aimed at replicating typical recreational exercise activities (i.e., ~40‐min brisk walk followed by light jogging) performed by lay individuals attending Foro Italico Umberto I in Palermo. While we did not measure hydration status prior or following each test in the field, the participant was required to drink at least ~500 mL of water prior to attending the tests, to ensure euhydration.

We used two commercially available Heat Stress Trackers (Kestrel 5400 WBGT, USA) to evaluate local meteorological conditions in both the shaded resting area (SHADE, location: 38°07′03.7″N 13°22′28.3″ E) as well as the area under direct sunlight (SUN, location: 38°07′03.9″ N 13°22′28.9″ E; Figure 1b), and we used the calculated wet‐bulb globe temperature (WBGT) as indicator of heat stress (Havenith & Fiala, 2015). Furthermore, we measured the participant's: (a) core temperature (*T*_core_) continuously via a gastrointestinal telemetric pill (BodyCap, France) ingested 3 h prior to testing; (b) local skin temperature at four sites (chest, deltoid, thigh and shin) via wireless thermistors (Maxim, USA); heart rate (HR) via a chest strap and watch (Polar, Finland); and whole‐body sweat losses (pre‐to‐post protocol change in body mass) with a high precision scale (SECA, Germany). Finally, we measured distance covered and speed during walking and running with a wearable Global Positioning System device (GPS) (Polar, Finland). Reporting for this case report followed the 2013 CARE Checklist guidelines (https://www.care‐statement.org/checklist).

## 3 RESULTS

**FIGURE 2:**
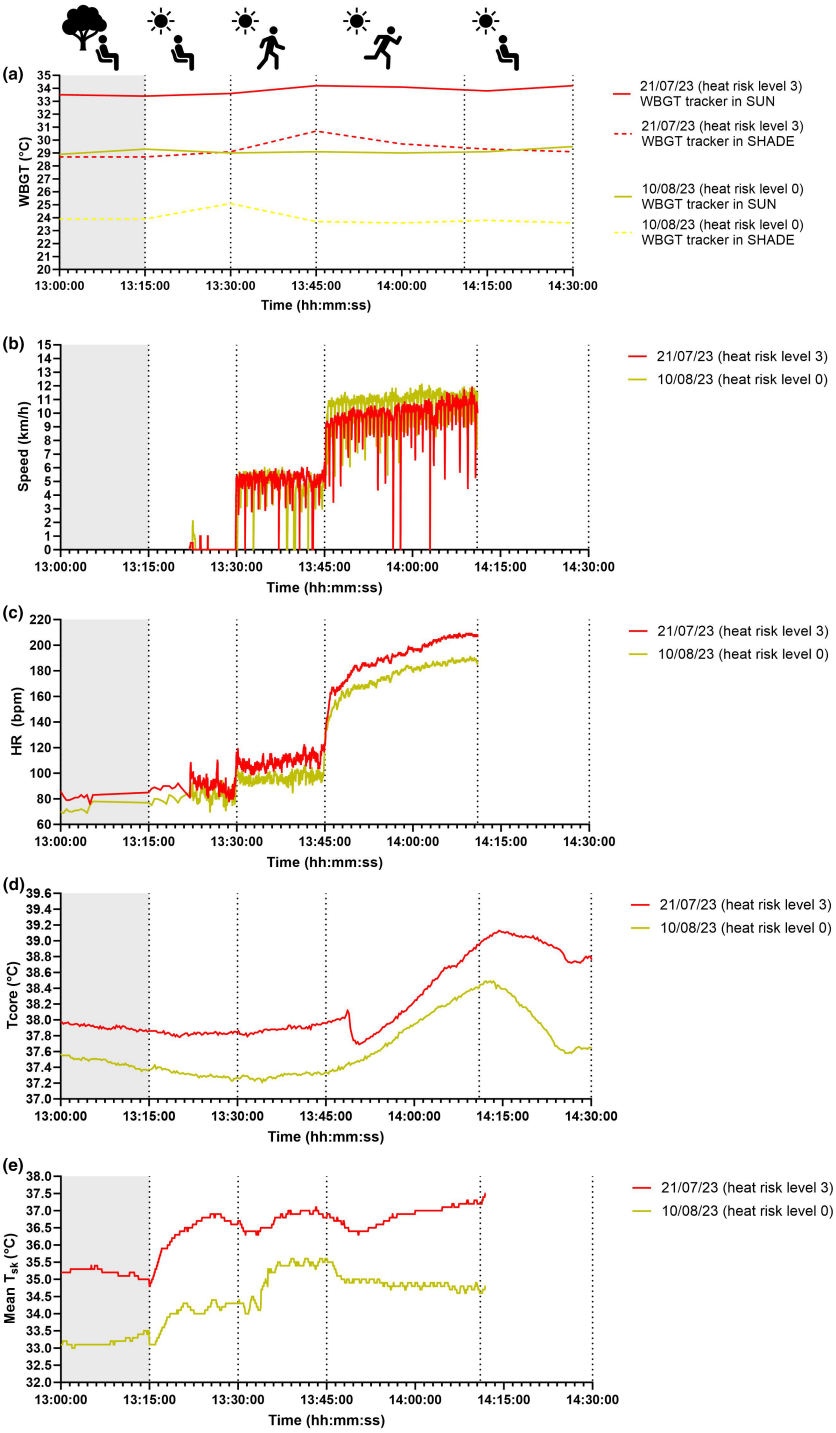
Time‐dependent changes in WBGT (a), walking and running speed (b), heart rate (c) core (d) and mean skin temperatures (e) during resting, walking, and light running cycles in the SHADE and SUN, as recorded on July 21 and August 10, 2023.

On July 21, average WBGT over the course of the 90‐min recording time was 33.8°C (SD 0.3°C) in SUN, and 29.3°C (SD 0.7°C) in SHADE (Figure 2a). Associated air temperature, globe temperature, relative humidity, and wind speed in SUN were 36.7°C (SD 0.8°C), 45.9°C (SD 0.5°C), 51.7% (SD 4.2%), and 0.7 m/s (SD 0.2 m/s), respectively. On August 10, average WBGT in SUN was 29.1°C (SD 0.2°C), and 23.9°C (SD 0.5°C) in SHADE (Figure 2a). Associated air temperature, globe temperature, relative humidity, and wind speed in SUN were 30.9°C (SD 0.4°C), 39.0°C (SD 4.6°C), 50.5% (SD 1.2%), and 0.7 m/s (SD 0.2 m/s), respectively.

By design, the rest and physical activity cycles resulted in the similar total distance covered (i.e., July 21: 6.6 km; August 10: 6.3 km); similar average speed (i.e., July 21: 7.2 km/h; August 10: 7.8 km/h; Figure 2b). Based on the fitness level of the participant, as well as on the previously laboratory‐established relationship between his walking/running speed and *V*O_2_, the walking and running speeds averaged during the field tests (i.e., ~5 and 10 km/h) corresponded to exercise intensities of ~41% and ~79% of the participant's *V*O_2max_, respectively.

Average and peak HR during the walk and running phases in SUN were higher during July 21 (walk: 109 ± 5 bpm; run: 191 ± 16 bpm; peak: 209 bpm; Figure 2c) than the August 10 test (walk: 96 ± 4 bpm; run: 174 ± 15 bpm; peak: 190 bpm). Due to technical reasons, HR monitoring was stopped at the end of the running phase.

Resting *T*_core_ was meaningfully higher during July 21 than the August 10 test at the end of the resting (37.84 vs. 37.27°C**)** and walking periods (37.96 vs. 37.34°C; Figure 2d) in SUN. *T*_core_ also raised to a higher absolute value during July 21 than the August 10 test at the end of running in SUN (38.96 vs. 38.44°C; Figure 2d), and it continued to increase following the termination of exercise (July 21, peak: 39.13°C; August 10, peak: 38.48°C). At the end of the 15‐min seating in SUN following the run, *T*_core_ remained largely elevated during the July 21 test only (i.e., 38.76 vs. 37.64°C, Figure 2d).

Mean *T*_sk_ was meaningfully higher during July 21 than the August 10 test at the end of the resting (36.7 vs. 34.3°C**)** and walking periods in SUN (36.8 vs. 35.6°C; Figure 2e). Mean *T*_sk_ also raised to a higher absolute value during July 21 than the August 10 test at the end of running in SUN (37.2 vs. 34.6°C; Figure 2d). Due to technical reasons, mean *T*_sk_ monitoring was stopped at the end of the running phase.

Whole‐body sweat losses corresponded to 1.7 and 2 kg during the July 21 and August 10 tests, respectively.

## 4 DISCUSSION

Our case report indicated that ~40 min of outdoor walking and light running in 33.8°C Wet Bulb Globe Temperature (WBGT) conditions (July 21; 1–3 pm) resulted in significant physiological stress in a healthy 37‐years male (i.e., peak heart rate: 209 bpm; core temperature: 39.13°C; mean skin temperature: 37.2°C; whole‐body sweat losses: 1.7 kg) exercising under environmental conditions below the laboratory‐tested limits of human adaptability to extreme heat (Vecellio et al., 2022). The observed physiological responses during Sicily's 2023 heatwave do justify the level 3 heat‐health alert issued in Palermo on July 21, 2023 (Regione Siciliana, 2023a); however, our data indicated that the heath‐health protection measures should have clearly extended beyond the most vulnerable individuals (as recommended in the alert) to also include younger, healthy groups who may have been at risk of heat illness if engaging in recreational exercise at the levels tested in this case study (i.e., 37‐years male with a *V*O_2max_ of 45 mL/min/kg exercising between 40% and 80% of their *V*O_2max_ for ~40 min).

Most importantly, significant physiological stress was also observed during less severe heat conditions (August 10, 1–3 pm; WBGT: 29.1°C; peak heart rate: 190 bpm; core temperature: 38.48°C; whole‐body sweat losses: 2 kg). The heat‐alert level issued for this day was the lowest possible (i.e., no health risks due to meteorological conditions Regione Siciliana, 2023b); hence, and on the basis of our field data, we argue that the alert level may have underestimated the thermal stress that individuals of varying ages would have encountered in Palermo on August 10, 2023.

Besides their applied relevance, and while acknowledging the nature of this case report (*N* = 1), our field measurements, in particular mean *T*_sk_ data, are in line with previously reported laboratory experiments that considered the role of solar radiation for ecologically‐valid predictions of the impact of heat on human physical work capacity (Foster et al., 2022). Specifically, our mean *T*_sk_ data during July 21 (i.e., 37.2°C) and August 10 (i.e., 34.6°C) are almost identical to the ones reported by Foster et al. (2022) at similar WBGT levels (i.e., see Foster et al., 2022 Figure 2c). Such mean *T*_sk_ values have been demonstrated to induce work capacity decays of up to 60% during heat exposure (Foster et al., 2022). While our experimental paradigm was conceived to replicate recreational exercise activities, due to the critical role of mean *T*_sk_ in predicting work capacity in the heat, our observations may also be relevant for occupational scenarios that are likely to be accompanied by sustained high mean *T*_sk_. Finally, we also acknowledge that our case report data provide further (field) confirmation to Vecellio et al.'s (2022) recent (laboratory) observations that *T*_sk_ typically exceeds 35°C after a short duration in ambient thermal environments above 36°C (see our Figure 2e). However, it is important to note that during the warm and humid conditions of July 21, under no instances the recorded mean *T*_sk_ exceeded *T*_core_, meaning that a consistent, positive thermal gradient for heat exchange was present between the core and the skin.

In conclusion, our field data highlight the urgent need to combine ecologically valid laboratory‐ and field‐based assessments of human responses to extreme heat (Ebi et al., 2021; Vanos et al., 2020) and humidity (Baldwin et al., 2023). This approach will be essential to inform the development of future, more realistic projections of heat‐related health risks across groups varying in age and clinical status.

## FUNDING INFORMATION

AV and SF were supported by PhD studentships from the University of Palermo, Italy.

## CONFLICT OF INTEREST STATEMENT

The authors report no financial or non‐financial interests that are directly or indirectly related to the work submitted for publication.

## ETHICS STATEMENT

One of the authors acted as the participant for the study, which received ethical approval from the University of Southampton Ethics Committee (ERGOII 91295).

## Data Availability

Raw data can be made available upon request to the corresponding author.
